# Severe Obesity Defined by Percentiles of WHO and Cardiometabolic Risk in Youth with Obesity

**DOI:** 10.3390/children11111345

**Published:** 2024-11-01

**Authors:** Giuliana Valerio, Procolo Di Bonito, Anna Di Sessa, Giada Ballarin, Valeria Calcaterra, Domenico Corica, Maria Felicia Faienza, Francesca Franco, Maria Rosaria Licenziati, Claudio Maffeis, Giulio Maltoni, Emanuele Miraglia del Giudice, Anita Morandi, Enza Mozzillo, Malgorzata Wasniewska

**Affiliations:** 1 Department of Medical, Movement Sciences and Wellbeing, University of Napoli “Parthenope”, 80133 Napoli, Italy; giada.ballarin@uniparthenope.it; 2 Department of Internal Medicine, “S. Maria delle Grazie” Hospital, 80078 Pozzuoli, Italy; procolodibonito53@gmail.com; 3 Department of Woman, Child and of General and Specialized Surgery, University of Campania “Luigi Vanvitelli”, 80138 Napoli, Italy; anna.disessa@unicampania.it (A.D.S.); emanuele.miraglia@unicampania.it (E.M.d.G.); 4 Department of Internal Medicine, University of Pavia, 27100 Pavia, Italy; valeria.calcaterra@unipv.it; 5 Department of Human Pathology in Adulthood and Childhood, University of Messina, 98122 Messina, Italy; domenico.corica@unime.it (D.C.); malgorzata.wasniewska@unime.it (M.W.); 6 Pediatric Unit, Department of Precision and Regenerative Medicine and Ionian Area, University of Bari “Aldo Moro”, 70124 Bari, Italy; mariafelicia.faienza@uniba.it; 7 Pediatric Department, Azienda Sanitaria Universitaria Friuli Centrale, Hospital of Udine, 33100 Udine, Italy; francesca.franco@asufc.sanita.fvg.it; 8 Neuro-Endocrine Diseases and Obesity Unit, Department of Neurosciences, Santobono-Pausilipon Children’s Hospital, 80129 Napoli, Italy; mrlicenziati@santobonopausilipon.it; 9 Department of Surgery, Dentistry, Pediatrics and Gynecology, Section of Pediatric Diabetes and Metabolism, University and Azienda Ospedaliera Universitaria Integrata of Verona, 37126 Verona, Italy; claudio.maffeis@univr.it (C.M.); anita.morandi@univr.it (A.M.); 10 Pediatric Unit, IRCCS Azienda Ospedaliero-Universitaria di Bologna, 40138 Bologna, Italy; giulio.maltoni@aosp.bo.it; 11 Section of Pediatrics, Department of Translational Medical Science, Regional Center of Pediatric Diabetes, University of Naples “Federico II”, 80131 Napoli, Italy; mozzilloenza@gmail.com

**Keywords:** body mass index z-score, cardiometabolic risk, severe obesity, international obesity task force, world health organization

## Abstract

**Background/Objectives:** The pediatric definition of severe obesity (OB) depends on the body mass index (BMI) references. We evaluated different BMI-derived metrics of the World Health Organization (WHO) system to define which cut-off is associated with the highest cardiometabolic risk (CMR); **Methods:** In this multicentric study, data were retrieved for 3727 youths (1937 boys; 2225 children, 1502 adolescents). OB was defined as BMI > 97th percentile (BMI_97th_), severe OB was defined as BMI > 99th percentile (BMI_99th_), BMI ≥ 120% of the 97th percentile (120% BMI_97th_), or BMI Z-score > 3 (WHO tables), or BMI ≥ the International Obesity Task Force (IOTF) value crossing a BMI of 35 kg/m^2^ at the age of 18 (IOTF_35_). The continuous CMR Z-score (sum of residual standardized for age and sex of waist-to-height ratio, systolic and diastolic blood pressure, triglycerides, and HDL-cholesterol x −1) and the cluster of at least two CMR factors (hypertension, high triglycerides, low HDL-cholesterol, and high waist-to-height ratio) were calculated. **Results:** Continuous CMR Z-score was significantly higher both in children or adolescents with severe OB defined by 120% BMI_97th_ compared to BMI_99th_ (*p* < 0.0001), while it was lower only in adolescents with severe OB defined by 120% BMI_97th_ compared to BMI Z-score >3 (*p* < 0.0001). Compared to 120% BMI_97th_, BMI Z-score > 3 and IOTF_35_ had higher specificity, but lower sensitivity in identifying children and adolescents with clustered CMR factors. **Conclusions:** The definition of severe OB based on 120% BMI_97th_ is superior to BMI_99th_ but it is inferior to BMI Z score > 3 as far as the association between severe OB and CMR factors is concerned. Pediatricians should take into consideration the implication of the use of different BMI metrics in those countries that recommend the WHO system. WHO BMI Z-score > 3 and IOTF_35_ can be used interchangeably to predict cardiometabolic risk.

## 1. Introduction

Severe obesity (OB) in adults is defined by a body mass index (BMI) value ≥ 40 kg/m^2^ [1]. It has been associated with a high risk of developing type 2 diabetes, hypertension, congestive heart failure, and poor therapeutic success [2]. In such cases, bariatric surgery currently represents the main therapeutic option able to induce significant weight loss and mitigation of cardiometabolic comorbidities including type 2 diabetes [3].

The definition of severe OB in children and adolescents is still a matter of debate and depends on the BMI reference values used. In Canada and several European countries, including Italy, the World Health Organization (WHO) growth reference is used [4]. According to this system, the definition is based either on BMI Z-score > 3 standard deviations (which corresponds to 99.9th percentile) [5] or BMI ≥ 99th percentile (as a rounded percentile of the 99.9th) [5,6]. On the contrary, the recent guideline for the treatment of obesity released by the American Academy of Pediatrics recommends using percentages of the 95th percentile of BMI for age and sex (BMI_95th_) to indicate different levels of severe OB [7]. In fact, the extreme values of BMI Z-score released by the Centers for Disease Control (CDC) are considered less reliable due to sparse reference data and compression > 97th percentile. Namely, the limit of BMI ≥ 120% of the CDC 95th percentile (BMI 95_th_) has been proposed to classify children and adolescents with severe OB [8,9]. In addition, the International Obesity Task Force (IOTF) system provides different age and sex BMI cut-offs to define severe OB, using smooth sex-specific BMI curves, constructed to match the values of 35 kg/m^2^ at 18 years (IOTF_35_) [10]. Since the cut-offs were developed on large data sets from six countries or regions covering different races/ethnicities, this system can be also used for international comparisons. 

The discrepancy in the categorization of severe OB may have consequences either in public health or in clinical practice. For instance, by using different definitions the classification of severe OB in children is heterogeneous, as shown by a recent systematic review with meta-analysis [11]. Therefore, a unified cut-off would provide a better comparison at international level and understanding of the clinical implications. Of note, the indication of bariatric surgery in adolescents is based on percentages of BMI_95th_ (namely BMI≥ 140% of the 95^th^ percentile, or BMI ≥ 120% of the 95th percentile with a comorbidity), or on absolute values of 40 and 35 kg/m^2^ [5], which does not have an equivalent in those countries using BMI Z-scores.

It is well known that cardiometabolic risk factors worsen with increasing severity of OB [12]. As far as we know, the empirical evidence for comparing the cardiometabolic risk across different BMI-derived metrics of the WHO tables, namely extended values of BMI over the 97th percentile and BMI Z-scores is lacking. Therefore, we compared individual and clustered cardiometabolic risk factors among different metrics of OB and severe OB, namely 97th percentile, 99th percentile, and BMI ≥ 120% of the 97th percentile (120% BMI_97th_) of the WHO tables in children and adolescents with OB. Furthermore, a comparison of the cardiometabolic variables between youths with BMI Z-score > 3, 120% BMI_97th_, and IOTF_35_ was performed. 

## 2. Materials and Methods

### 2.1. Study Design and Participants

We retrospectively analyzed the data of children and adolescents retrieved from the database of the CARITALY (CARdiometabolic risk factors in overweight and obese children in ITALY) study, a multicenter cross-sectional study on the behalf of the Obesity study group of the Italian Society for Pediatric Endocrinology and Diabetology [13]. Participants were observed between 2003–2020 in ten Italian centers for the management of pediatric obesity distributed across different regions. The inclusion criteria were age 6–17 years, BMI > 97th percentile of the WHO tables, and having complete anthropometric, clinical, and biochemical variables in the data set. The exclusion criteria were secondary OB, type 1 or type 2 diabetes, LDL-C ≥ 190 mg/dL, TGs ≥ 400 mg/dL, any other chronic disease, or chronic use of drugs leading to metabolic disturbances (such as steroids).

### 2.2. Anthropometric Assessement

In each center, physical examinations were performed by trained pediatricians and pubertal maturation was evaluated according to Tanner staging. Anthropometric parameters were measured following standard procedures [13]. BMI was calculated as weight (kg)/height (m)^2^; BMI Z-scores were calculated using the WHO AnthroPlus software v.1.0.4 [14]. Waist circumference was measured at the midpoint between the last rib and the iliac crest at minimal respiration when the participant was in a standing position. The waist-to-height ratio (WHtR) was calculated by dividing waist circumference (cm) by height (cm). 

### 2.3. Clinical and Laboratory Evaluations

Blood pressure (BP) was measured three times within two min in the sitting position after 5 min rest through an aneroid sphygmomanometer with an appropriately sized cuff, as elsewhere described [13]. 

Blood samples for glucose, insulin, triglycerides (TGs), cholesterol, and high-density lipoprotein-cholesterol (HDL-C) were drawn after an overnight fast. These biochemical parameters were analyzed in the centralized laboratory of each center, as elsewhere described [13]. 

All laboratories belonged to the Italian National Health System and were certified according to International Standards ISO 9000 [15] with semi-annual quality controls and inter-lab comparisons.

### 2.4. Definition of Obesity and Severe Obesity

Obesity was defined as BMI > 97th percentile (BMI_97th_). Severe OB was defined according to the following BMI thresholds: 99th percentile (BMI_99th_), ≥120% BMI_97th_, BMI Z-score > 3 of the WHO tables, or BMI > the IOTF value plotted on the sex-related curves crossing a BMI of 35 kg/m^2^ at the age of 18 years (IOTF_35_). 

### 2.5. Definitions of Cardiometabolic Risk

The continuous metabolic risk score (cCMR) was expressed as Z-score and calculated by linear regression analysis. The cCMR was obtained using the sum of residual standardized for age and sex of WHtR, systolic BP, diastolic BP, TGs (Ln), and HDL-C x −1. 

In addition, the cardiometabolic risk was analyzed as a categorical variable, based on a cluster of at least two factors among hypertension, high TGs, low HDL-C, and high WHtR. Hypertension was defined according to the criteria of European Society of Cardiology based on 95th percentile of systolic and/or diastolic BP for age, sex, and height [16]. High TGs was defined by a value above 100 mg/dL in children and 130 mg/dL in adolescents. Low HDL-C was defined by HDL-C levels < 40 mg/dL. High WHtR was defined by a value ≥ 0.65 [17].

### 2.6. Statistical Analyses

The normal distribution of the variables was assessed by using the Kolmogorov–Smirnov test. Continuous variables with normal distribution were expressed as mean ± standard deviation. Variables with skewed distribution were log transformed using natural log (Ln) for statistical analyses, but they were presented as median and interquartile range for a better understanding. The sample was stratified into three mutually exclusive groups according to the following BMI cut-offs: BMI_97th_, BMI_99th_, and 120%BMI_97th_. Comparisons among continuous variables were assessed by ANOVA with the post hoc Bonferroni correction. Qualitative variables were presented as number and percentage (%) and compared using the χ^2^ test. 

The diagnostic accuracy of either BMI Z-score > 3, 120% BMI_97th_, or IOTF_35_ to discriminate the presence of clustered cardiometabolic risk factors was assessed. Receiver operating characteristic (ROC) curve analysis was used to evaluate accuracy (area under the curve), specificity, sensitivity, positive predictive value (PPV), and negative predictive value (NPV), using 2 × 2 tables.

A *p* value < 0.05 was considered for statistical significance. Statistical analyses were conducted using IBM SPSS Statistics software, Version 28.0 (Armonk, NY, USA IBM corporation).

## 3. Results

The sample was represented by 3727 youths (1937 boys and 1790 girls), divided into 2225 children (age < 12 years) and 1502 adolescents (age ≥ 12 years). Mean age was 11.2 ± 2.4 years (range 6–17 years). 

The characteristics of the three mutually exclusive categories based on different BMI-derived metrics are described in Table 1. Anthropometric, clinical, and biochemical variables progressively increased from the lowest to the highest BMI category, while HDL-C levels decreased (*p* < 0.0001 for all). No difference among groups was observed only for fasting glucose and total cholesterol.

**Figure 1:**
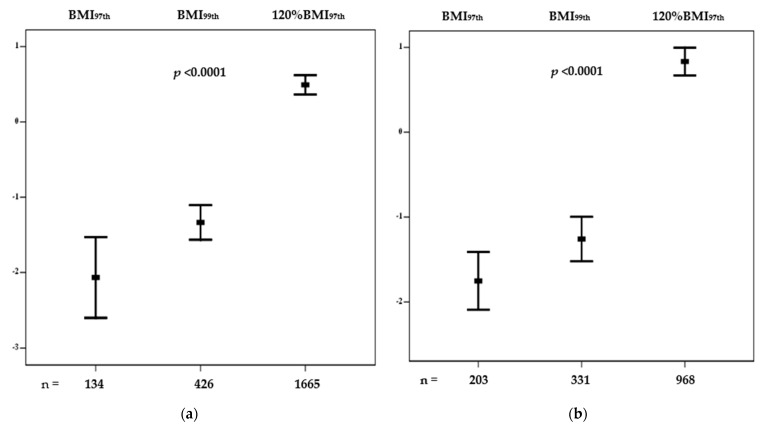
Continuous cardiometabolic risk Z-score in children (**a**) and adolescents (**b**) by different BMI-derived metrics. Abbreviations: BMI_97th_, BMI > 97th percentile; BMI_99th_, BMI > 99th percentile; 120% BMI_97th_, BMI ≥ 120% of the 97th percentile.

Mean values of cCMR Z-score stratified in the three BMI categories are shown in Figure 1 separately by age group (children, panel (a); adolescents, panel (b)). Differences between categories were significant for all groups (*p* < 0.0001), either in children or adolescents. 

Comparisons among groups with severe OB classified by BMI Z-score > 3, 120% BMI_97th_, or IOTF_35_ are shown separately by age group in Table 2 (children) and Table 3 (adolescents). The prevalence of severe OB as defined by the cut-off of 120% BMI_97th_ was significantly higher than that observed by using BMI Z-score > 3 or IOTF_35_, both in children (74.8% vs. 60.5% or 57.7%) and adolescents (64.4% vs. 37.7% or 39.7%), *p* < 0.01. No significant difference was found between BMI Z-score > 3 or IOTF_35_ definitions.

Children with severe OB defined by 120% BMI_97th_ showed lower values of BMI, BMI Z-score, and WHtR than those defined by BMI Z-score > 3 and IOTF_35_, while they showed no difference with regard to individual cardiometabolic risk factors, except for a lower cCMR Z-score compared to IOTF_35_ (*p* < 0.01) (Table 2). Similar findings were found in adolescents with severe OB defined by 120% BMI_97th_, in whom cCMR Z-score was significantly higher than that found in the other two groups (Table 3). 

**Figure 2:**
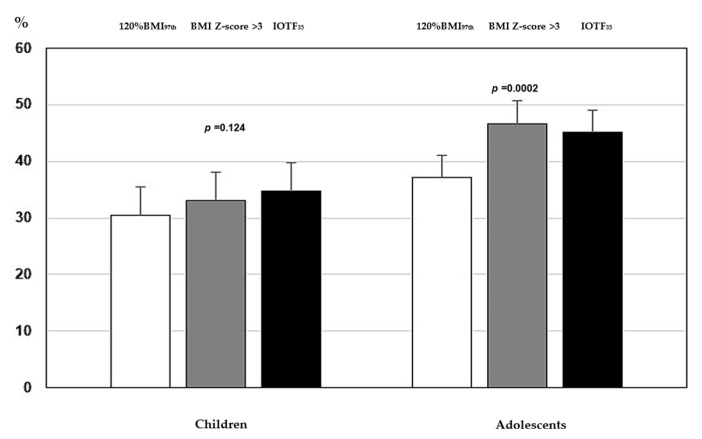
Proportion of youths with a cluster of cardiometabolic risk factors by 120% BMI_97th_ (white bars), BMI Z-score > 3 (grey bars), and IOTF_35_ (black bars) in children and adolescents. Abbreviations: 120% BMI_97th_, BMI ≥ 120% of the 97th percentile; IOTF_35_, IOTF value plotted on the sex-related curves crossing a BMI of 35 kg/m^2^ at the age of 18 years.

Similarly, the prevalence of clustered cardiometabolic risk factors was lower in adolescents with severe OB classified by 120% BMI_97th_ compared to BMI Z-score > 3 or IOTF_35_ (*p* < 0.001), while no substantial difference was found among the three classification methods in children (Figure 2).

AUC, sensitivity, specificity, PPV, NPV of 120% BMI_97th_, BMI Z-score > 3, or IOTF_35_ defined categories of severe OB for predicting clustered cardiometabolic risk are synthetized in Table 4. The AUC values ranged between 0.60 and 0.65 in children, and 0.62 and 0.66 in adolescents. Compared to 120% BMI_97th_, BMI Z-score > 3 and IOTF_35_ had higher specificity and PPV, but lower sensitivity and NPV in identifying children and adolescents with clustered cardiometabolic risk factors. 

## 4. Discussion

The present study provided the empirical evidence that cardiometabolic risk factors increased with rising degrees of OB, as defined by BMI-derived metrics from the WHO system; specifically, the threshold of 120% BMI_97th_ had a stronger association with clustered risk factors compared to BMI_99th_ in children and adolescents. Furthermore, compared to 120% BMI_97th_, BMI Z-score > 3 was associated with worse adiposity measures both in children and adolescents, although neither definition was significantly associated with individual cardiometabolic risk factors. On the contrary, adolescents with severe OB defined by BMI Z-score > 3 had worse cCMR scores and higher prevalence of clustered cardiometabolic risk factors than those defined by BMI ≥ 120% of 97th percentile. No difference was found between BMI Z-score > 3 and IOTF_35_, another classification used for international comparisons.

Severe OB affects a large number of children in Europe [18]. Between 2007–2013, the prevalence of severe OB in 6- to 9-year-old school children from 21 countries of the WHO European Region ranged from 1.0% to 5.5% (WHO definition), with the highest levels (above 4%) in Southern Europe, including Italy. 

As it emerged from a recent systematic review on the global prevalence of severe OB in childhood, several definitions have been used in children and adolescents [11]. In particular, BMI ≥ 120% of the 95th percentile, as proposed by the CDC and endorsed by the American Academy of Pediatrics [7], and BMI ≥ 35 kg/m^2^ were the most frequently used. Other definitions relied upon the cut-off of age- and gender-specific 99th percentile from the CDC or WHO standards, BMI > 3 SDs from the WHO system, or cut-off values according to the IOTF system. 

The drawbacks of using BMI Z-score for very high BMIs from the CDC system led some countries to adopt the 120% of the 95th percentile of BMI. On the contrary, the BMI Z-score is still in use in those countries that adhered to the WHO definition, since it does not suffer from the limitations associated with CDC [4]. In fact, although both CDC and WHO used the same database from the US National Center for Health Statistics [19], some manipulations were performed to build the WHO standards: measurements taken after 1973 were truncated to avoid the obesity epidemic and 3% of children with ‘unhealthy’ weights for heights were excluded. In addition, the SD23 restriction of the LMS (Lambda, Mu, and Sigma) method (i.e., the standard deviation at each age was fixed to the distance between ±2 SDs and ±3 SDs) was applied for data beyond the limits of the observed values (i.e., between −3 SDs and 3 SDs) [4]. 

Previous cross-sectional studies reported that the prevalence of several cardiometabolic risk factors, such as dyslipidemia, hypertension, type 2 diabetes, and fatty liver disease was higher i8 individuals with severe OB compared to mild OB with any of the definitions used [12,20,21,22]. In agreement with previous studies, anthropometric, clinical, and individual cardiometabolic risk factors (except for fasting glucose and total cholesterol) progressively worsened from the lowest to the highest BMI category, as represented by 120% BMI_97th_. In particular, the cCMR score was greatly higher both in children and adolescents with severe OB defined by 120% BMI_97th_ compared to BMI_99th_. These results confirm a previous study conducted by our group in a different cohort of youths with OB, where the definition of severe OB slightly varied from the definition adopted in the present study (respectively, 120% BMI_95th_ instead of 120% BMI_97th_) [23]. 

To make the situation more complicated, the WHO definition of severe OB can be alternatively based on BMI_99th_ or BMI_99.9th_ (equivalent to three standard deviations). Therefore, we also compared anthropometric, clinical, and individual cardiometabolic risk factors between 120% BMI_97th_ and BMI Z-score > 3. Although the prevalence of severe OB was significantly higher when the cut-off of 120% BMI_97th_ was compared to BMI Z-score > 3 both in children (69.7% vs. 56.4%) and adolescents (63.9% vs. 37.4%), both systems appeared to be interchangeable in children, without a particular superiority of one to another in stratifying individuals at cardiometabolic risk. Instead, in adolescents, worse cCMR scores and higher prevalence of clustered cardiometabolic risk factors were found in individuals with severe OB defined by BMI Z-score > 3 compared to 120% BMI_97th_. Interestingly, no difference was found between BMI Z-score > 3 and IOTF_35_ in the anthropometric, clinical, and biochemical variables, or in the cCMR Z-score.

The modest AUC values (0.60–0.66) obtained by ROC analysis indicate a relatively low discriminatory power for BMI Z-score > 3, 120% BMI_97th_, and IOTF_35_ in predicting cardiometabolic risk factors (AUCs ranging from 0.60 for 120% BMI_97th_ in children to 0.66 for BMI Z-score > 3 in adolescents) (Table 4). Remarkably, it was quite similar to that reported by Ball et al. in a large sample of children and adolescents enrolled in the Canadian Pediatric Weight Management Registry (AUC 0.62 for BMI Z-score and 0.64 for 120% BMI_97th_) [24]. This finding confirms the limited utility of any BMI-derived metrics as proxy measures of cardiometabolic outcomes in youth with severe OB. However, even other anthropometric measurements of abdominal adiposity, such as waist circumference or WHtR, exhibited similar performance in discriminating youths with cardiometabolic risk factors [25]. In our sample, BMI Z-score > 3 and IOTF_35_ had higher specificity and PPV, but lower sensitivity and PPN than 120% BMI_97th_ in identifying individuals with clustered cardiometabolic risk factors, especially in adolescents. Therefore, both WHO BMI Z-score > 3 and IOTF_35_ cut-offs can be used as most stringent criteria for the definition of cardiometabolic outcome associated to severe OB.

Our study is not exempt from having some limitations. Youths with OB were recruited in pediatric obesity services; therefore, findings cannot be extended to the general population. However, it should be underlined that previous studies that analyzed nationally representative data also demonstrated that severe OB was associated with an increased prevalence of cardiometabolic risk factors compared to overweight and milder forms of OB [12]. Moreover, other factors that might influence cardiometabolic risk, such as genetics, perinatal and early life determinants, lifestyle habits, and fat distribution were not assessed in the present study. In addition, the investigation was limited to cardiometabolic risk factors, without considering the respiratory, musculoskeletal, or psychosocial complications associated with severe OB. Lastly, the cross-sectional design of the study does not allow to establish causality between BMI metrics and cardiometabolic outcomes. 

## 5. Conclusions

Our results show that within the WHO system, the definition of severe OB based on 120% of 97th percentile is superior to BMI_99th_ but it is inferior to BMI Z score > 3 as far as the association between severe OB and cardiometabolic risk factors is concerned. The definition based on BMI Z-score > 3 has a discriminatory advantage over of the 120% BMI_97th_ for identifying severely obese children at increased cardiometabolic risk, particularly in adolescents. However, the higher sensitivity of the 120% BMI_97th_ definition in identifying a higher number of children and adolescents with severe OB at expense of lower specificity may provide advantages in terms of early cardiovascular prevention. 

Pediatricians should take the into consideration the implication of the use of BMI_99th_ or BMI Z-score > 3 in those countries that recommend using the WHO growth reference. Within the limitation of using BMI, BMI Z-score > 3 can be used as a more stringent criterion for the prediction of cardiometabolic outcome associated to severe OB also when compared to BMI ≥ 120% of the 97th percentile. WHO BMI Z-score > 3 and IOTF_35_ can be used interchangeably to predict cardiometabolic risk.

## Figures and Tables

**Table 1:** Anthropometric, clinical, and biochemical variables by different BMI-derived metrics among children and adolescents with obesity (BMI_97_**_th_**) and severe obesity (BMI_99_**_th_** or 120% BMI_97th_).

	BMI_97th_	BMI_99th_	120%BMI_97th_	*p* Value
n = 3727	337	757	2633	
Male gender, n (%)	124 (36.8)	339 (44.8)	1474 (56.0)	<0.0001
Prepubertal stage, n (%)	42 (12.5)	188 (24.8)	828 (31.4)	<0.0001
Age (years)	12.4 ± 2.4	11.4 ± 2.5	11.0 ± 2.4	<0.0001
BMI, kg/m^2^	25.5 ± 2.7	26.5 ± 3.0	31.1 ± 4.6	<0.0001
BMI Z-score	2.1 ± 0.1	2.6 ± 0.2	3.5 ± 0.6	<0.0001
WHtR	0.57 ± 0.06	0.59 ± 0.05	0.64 ± 0.06	<0.0001
Glucose, (mg/dL)	87.3 ± 7.8	86.5 ± 8.4	86.7 ± 9.1	0.594
Insulin, (µUI/mL)	13.4 (9.5–19.1)	13.0 (9.1–18.6)	16.4 (11.7–24.1)	<0.0001
Cholesterol, (mg/dL)	160.1 ± 33.7	156.5 ± 30.6	157.3 ± 30.0	0.492
HDL-C (mg/dL)	50.7 ± 12.7	49.3 ± 11.1	47.1 ± 10.6	<0.0001
Triglycerides (mg/dL)	75.0 (55.0–103.0)	73.0 (55.0–98.0)	83.0 (62.0–112.0)	<0.0001
Systolic BP (mmHg)	109.8 ± 13.7	109.6 ± 13.0	112.7 ± 14.1	<0.0001
Diastolic BP (mmHg)	65.5 ± 10.0	65.2 ± 9.3	67.3 ± 10.0	<0.0001

Data are expressed as mean ± standard deviation, median (IQ range), n (%). Abbreviations: BMI_97th_, BMI > 97th percentile; BMI_99th_, BMI > 99th percentile; 120% BMI_97th_, BMI ≥ 120% of the 97th percentile; BP, blood pressure; HDL-C, high-density lipoprotein-cholesterol; WHtR, waist-to-height ratio.

**Table 2:** Comparison among children with severe obesity classified by 120% BMI_97th_**,** BMI Z-score > 3, or IOTF_35_.

	120% BMI_97th_	BMI Z-Score > 3	IOTF_35_
n = 2225	1665 (74.8%)	1346 (60.5%)	1284 (57.7%)
Male gender, n (%)	882 (53.0) ^a^	765 (56.8) ^b^	600 (46.7)
Age (years)	9.5 ± 1.5 ^c^	9.3 ± 1.5	9.3 ± 1.6
BMI, kg/m^2^	29.1 ± 3.4 ^c^	29.5 ± 3.6	29.6 ± 3.6
BMI Z-score	3.6 ± 0.6 ^c^	3.8 ± 0.5	3.8 ± 0.6
WHtR	0.64 ± 0.06 ^c^	0.65 ± 0.06	0.65 ± 0.06
Glucose, (mg/dL)	86.0 ± 8.6	85.8 ± 8.6	85.6 ± 8.7
Insulin, (µUI/mL)	14.7 (10.5–20.8)	14.9 (10.7–21.0)	15.0 (10.8–21.5)
Cholesterol, (mg/dL)	158.4 ± 29.2	158.2 ± 29.1	157.7 ± 29.3
HDL-C (mg/dL)	48.1 ± 10.7	47.9 ± 10.7	47.5 ± 10.7
Triglycerides (mg/dL)	81.0 (61.0–109.0)	82.0 (61.0–109.0)	82.0 (62.0–111.1)
Systolic BP (mmHg)	109.1 ± 13.1	108.9 ± 13.4	108.6 ± 13.4
Diastolic BP (mmHg)	65.4 ± 9.5	65.4 ± 9.6	65.4 ± 9.6
cCMR Z-score	0.49 ± 2.65 ^d^	0.66 ± 2.70	0.76 ± 2.62

Data are expressed as mean ± standard deviation, median (IQ range), n (%). Abbreviations: 120% BMI_97th,_ BMI ≥ 120% of the 97th percentile; BP, blood pressure; cCMR, continuous cardiometabolic risk; HDL-C, high-density lipoprotein-cholesterol; IOTF, International Obesity Task Force; IOTF_35_, IOTF value plotted on the sex-related curves crossing a BMI of 35 kg/m^2^ at the age of 18 years; WHtR, waist-to-height ratio. ^a^ *p* < 0.01, 120% BMI_97th_ vs. IOTF_35_; ^b^ *p* < 0.01, BMI Z-score > 3 vs. IOTF_35_; ^c^ *p* < 0.01, 120% BMI_97th_ vs. BMI Z-score > 3 and IOTF_35_; ^d^ *p* < 0.01, 120% BMI_97th_ vs. IOTF_35_.

**Table 3:** Comparison between adolescents with severe obesity classified by 120% BMI_97th_**,** BMI Z-score > 3, or IOTF_35_.

	120% BMI_97th_	BMI Z-Score > 3	IOTF_35_
n = 1502	968 (64.4%)	567 (37.7%)	597 (39.7%)
Male gender, n (%)	592 (61.2)	334 (58.9)	335 (56.1)
Age (years)	13.5 ± 1.4	13.6 ± 1.3	13.6 ± 1.3
BMI, kg/m^2^	34.5 ± 4.4 ^a^	37.0 ± 3.9	36.8 ± 3.8
BMI Z-score	3.3 ± 0.53 ^a^	3.6 ± 0.50	3.5 ± 0.50
WHtR	0.65 ± 0.07 ^a^	0.67 ± 0.07	0.67 ± 0.07
Glucose, (mg/dL)	87.9 ± 9.8	87.5 ± 10.2	87.8 ± 10.1
Insulin, (µUI/mL)	19.7 (14.4–29.2)	21.4 (15.3–32.3)	21.4 (15.7–32.2)
Cholesterol, (mg/dL)	154.9 ± 30.0	155.9 ± 31.2	155.1 ± 30.6
HDL-C (mg/dL)	45.5 ± 10.1	44.7 ± 10.0	44.6 ± 9.7
Triglycerides (mg/dL)	87.0 (65.0–115.0)	88.0 (65.0–117.0)	87.0 (65.0–117.0)
Systolic BP (mmHg)	118.9 ± 13.5	120.1 ± 13.8	120.3 ± 13.7
Diastolic BP (mmHg)	70.6 ± 10.2	71.4 ± 10.4	71.6 ± 10.5
cCMR Z-score	0.83 ± 2.59 ^a^	1.47 ± 2.60	1.43 ± 2.59

Data are expressed as mean ± standard deviation, median (IQ range), n (%). Abbreviations: 120% BMI_97th_, BMI ≥ 120% of the 97th percentile; BP, blood pressure; cCMR, continuous cardiometabolic risk; HDL-C, high-density lipoprotein-cholesterol; IOTF, International Obesity Task Force; IOTF_35_, IOTF value plotted on the sex-related curves crossing a BMI of 35 kg/m^2^ at the age of 18 years; WHtR, waist-to-height ratio. ^a^ *p* < 0.01, 120% BMI_97th_ vs. BMI Z-score > 3 and IOTF_35_.

**Table 4:** Performance of 120% BMI_97th_, BMI Z-score > 3, and IOTF_35_ in relation to a cluster of cardiometabolic risk factors.

	AUC	Sensitivity	Specificity	PPV	NPV
**Children**					
120% BMI_97th_	0.60 (0.58–0.63)	0.90 (0.89–0.92)	0.30 (0.29–0.32)	0.31 (0.29–0.32)	0.90 (0.89–0.92)
BMI Z-score > 3	0.63 (0.60–0.65)	0.80 (0.78–0.81)	0.46 (0.44–0.48)	0.33 (0.31–0.35)	0.87 (0.85–0.88)
IOTF_35_	0.65 (0.62–0.67)	0.79 (0.78–0.81)	0.50 (0.48–0.52)	0.35 (0.33–0.37)	0.88 (0.86–0.89)
**Adolescents**					
120% BMI_97th_	0.62 (0.59–0.65)	0.81 (0.79–0.83)	0.43 (0.40–0.45)	0.37 (0.35–0.40)	0.85 (0.83–0.86)
BMI Z-score > 3	0.66 (0.63–0.69)	0.60 (0.57–0.62)	0.72 (0.69–0.74)	0.47 (0.44–0.49)	0.81 (0.79–0.83)
IOTF_35_	0.65 (0.62–0.68)	0.61 (0.58–0.63)	0.69 (0.67–0.71)	0.45 (0.43–0.48)	0.81 (0.79–0.83)

Abbreviations: 120% BMI_97th_, BMI ≥ 120% of the 97th percentile, IOTF_35_, IOTF value plotted on the sex-related curves crossing a BMI of 35 kg/m^2^ at the age of 18 years; AUC, area under curve; PPV, positive predictive value; NPV, negative predictive value.

## Data Availability

The data presented in this study are available on request from the corresponding author. The data are not publicly available due to privacy concerns.
